# Agreement Between Two Swept-Source Optical Coherence Tomography Devices in Assessing Glistening on the Intraocular Lens In Vivo

**DOI:** 10.3390/diagnostics16050733

**Published:** 2026-03-01

**Authors:** Teresa Serrano González-Peramato, José Ignacio Fernández-Vigo, Beatriz De Pablo Gómez de Liaño, Ignacio Almorín-Fernández-Vigo, Lucía De Pablo Gómez de Liaño, Inés Sánchez-Guillén, Irene Serrano García, Ana Macarro-Merino, José Ángel Fernández-Vigo

**Affiliations:** 1 Department of Ophthalmology, Hospital Universitario Clínico San Carlos, Instituto de Investigación Sanitaria (IdISSC), 28040 Madrid, Spain; teresasgp@gmail.com; 2 Centro Internacional de Oftalmología Avanzada, 28010 Madrid, Spain; 3 Department of Immunology, ENT, and Ophthalmology, School of Medicine, Universidad Complutense de Madrid, 28040 Madrid, Spain; 4 Department of Ophthalmology, Hospital Central de la Cruz Roja San José y Santa Adela, 28003 Madrid, Spain; 5 Centro Internacional de Oftalmología Avanzada, 06010 Badajoz, Spain; 6 Department of Ophthalmology, Hospital Universitario 12 de Octubre, 28041 Madrid, Spain; 7 Department of Ophthalmology, School of Optics, Universidad Complutense de Madrid, 28040 Madrid, Spain; 8 Department of Ophthalmology, Hospital Universitario Perpetuo Socorro, 06010 Badajoz, Spain; 9 Unidad de Apoyo Metodológico a la Investigación, Hospital Clínico San Carlos, IdISSC, 28040 Madrid, Spain; 10 Department of Ophthalmology, School of Medicine, Universidad de Extremadura, 06006 Badajoz, Spain

**Keywords:** glistening, intraocular lens, optical coherence tomography, pseudophakic eye, cataract surgery

## Abstract

**Background/Objectives**: To analyze the agreement between two swept-source optical coherence tomography (SS-OCT) devices in assessing glistening on intraocular lenses (IOL). **Methods**: Patients who had previously undergone cataract surgery were included. They were sequentially examined using two SS-OCT devices: Anterion (Heidelberg Engineering Inc., Heidelberg, Germany) and Triton (Topcon, Inc., Tokyo, Japan). Six corresponding scans from both devices were compared, and glistening, observed as hyperreflective foci (HRF), was manually counted. The total number of HRF and the degree of glistening were measured and categorized into four groups. The agreement between the two devices was analyzed using the intraclass correlation coefficient (ICC). **Results**: A total of 333 eyes from 285 patients were evaluated. The mean age was 76.5 ± 8.0 years (range: 45–95). The median number of HRF detected in a single scan was 1.1 (IQR 0.0–10.2, range 0–176) using Triton and 2.7 (IQR 0.2–20.1, range 0–250) using Anterion. The ICC across different scans ranged from 0.8 to 0.9, indicating strong agreement between the two devices. Bland–Altman plots showed better concordance in lenses with low glistening grades, while higher grades revealed greater discrepancies, with Anterion detecting significantly more HRF than Triton. Among all factors studied, only postsurgical time was associated with glistening. **Conclusions**: Two different SS-OCT devices can detect and quantify glistening in IOLs. The concordance between them was high, particularly for lower glistening grades. However, in higher grades, Anterion detected significantly more HRF than Triton.

## 1. Introduction

Glistening in intraocular lenses (IOL) refers to the appearance of small, shiny, white or yellow spots, ranging from 1 to 20 µm. These spots correspond to fluid-filled microvacuoles (MV) within the IOL’s optic following implantation, due to interaction with the aqueous environment [1,2]. The main visual effect of glistening is increased intraocular light scatter [3], which has been shown to correlate with the number of MVs [4,5,6,7]. Importantly, the scatter perceived by the patient is estimated to be approximately 300 times greater than the backscattered light observed by the clinician during IOL examination [8].

Early methods for quantifying these MVs primarily involved their visualization during slit-lamp examinations. The characteristic glistening appearance results from the difference in refractive indices between water and the IOL polymer [9,10]. Several research groups have employed this approach to quantify hyperreflective foci (HRF) or spots, developing various classification scales for glistening [11,12,13,14,15,16,17,18,19]. However, this method requires considerable photographic skill to capture valuable images of the glistening phenomenon, which are then counted. Additionally, this process often involves post-processing of the images, so many researchers still rely on subjective assessments during slit-lamp examinations to detect and quantify IOL glistening [20].

Recently, the need for an objective and reproducible method to evaluate glistening has brought optical coherence tomography (OCT) to the forefront as a promising alternative. OCT is preferred for its speed, reproducibility, and independence from operator expertise. Werner et al. were the first to detect glistening using this technique through an ex vivo analysis of five acrylic hydrophobic IOLs that had been explanted due to complications. They found that OCT could help analyze the location and density of glistening [21], although their sample size was minimal. Our group recently conducted the first large-scale cross-sectional study to analyze IOLs in vivo using OCT, involving 150 subjects and employing the DRI-Triton^®^, a Swept-Source OCT device (SS-OCT, Topcon Corporation, Tokyo, Japan) [22]. Our results demonstrated that IOL glistening can be effectively identified, quantified, and classified using this OCT. Following this, we presented a study of 325 eyes in which we assessed glistening severity across four IOL models, demonstrating the ability to evaluate multiple lens types using SS-OCT with a deep-learning algorithm for a simple and objective evaluation [23].

Currently, several OCT models available on the market show potential for quantifying glistening, with two of the most widely used being the DRI-Triton and Anterion devices. Both systems employ swept-source technology, an advanced imaging method that utilizes longer wavelengths to achieve deeper penetration into intraocular tissues, enabling more comprehensive visualization and analysis. This technique also employs rapid wavelength variation to accelerate image acquisition and is less sensitive to displacement and eye movements, yielding more stable and precise images. Several recent studies have compared the performance of different SS-OCT devices in analyzing various ocular structures and calculating biometric parameters [24,25,26,27,28,29,30,31]. However, to date, no research has specifically evaluated the capability of these devices to detect and quantify glistening in IOLs. This gap highlights the need for further investigation into their potential application.

Therefore, this study aims to measure glistening using two SS-OCT devices, DRI-Triton and Anterion, and to assess the level of agreement between their measurements.

## 2. Materials and Methods

### 2.1. Patients

This observational cross-sectional study was conducted in 333 eyes (162 right and 171 left) from 285 individuals (119 males and 166 females). Patients were recruited consecutively among those visiting the Centro Internacional de Oftalmología Avanzada (Madrid, Spain) who came for a routine examination, including refraction measurements from 1 October 2023 to 30 November 2023.

The study protocol adhered to the tenets of the Declaration of Helsinki and was approved by the Institutional Review Board (IRB) at the University of Extremadura (Badajoz, Spain) before study initiation. Patients who met all inclusion and no exclusion criteria were enrolled after providing written informed consent.

After recording each subject’s medical history, the subjects underwent a comprehensive eye examination. Subjects were included if they were pseudophakic. Patients were excluded if they had conditions that could affect the occurrence of glistening (such as diabetes, glaucoma, uveitis, or inflammatory diseases). Additionally, patients were excluded if they had IOLs with decentration or abnormal positioning, central corneal opacities compromising media transparency, posterior segment conditions associated with poor fixation, or mental conditions that impeded adequate cooperation. The studied population had undergone cataract surgery between June 2005 and October 2023. The IOL models studied are detailed in Table 1.

All implanted lenses were hydrophobic acrylic (methacrylate copolymer) lenses manufactured by Alcon (Fort Worth, TX, USA). Eyes with unknown IOL models (*n* = 19; 5.7%) were excluded from the IOL model–glistening association analysis to ensure the accuracy and interpretability of the results.

### 2.2. Examination Protocol

In a standard examination, the data recorded for each participant included a complete medical history, including sex and age, visual acuity, and slit-lamp biomicroscopy. All OCT images were acquired after pupil dilation with the subjects sitting upright. Fixation was guided by a fixation light for all devices. In each subject, two OCT devices were employed to assess glistening: Triton (Topcon, Inc., Tokyo, Japan) and Anterion (Heidelberg Engineering Inc., Heidelberg, Germany). Examinations were performed on the same day by the same examiner (T.S.G-P.). The order in which the two examinations were performed was randomized.

The Anterion system is an SS-OCT system that uses a central wavelength of 1300 nm, with an axial resolution of <10 µm and a transverse resolution of 30 µm, acquiring 50,000 axial scans per second. The “metrics” option was employed, which provides six scans centered on the pupil. The device provides acquisition quality metrics in both programs, assessing motion, fixation, tear film and lid position during image capture. Only images with a positive quality rating (classified by the device as “pass”) were considered for evaluation.

The Triton system is an SS-OCT system that uses a central wavelength of 1050 nm, with an axial resolution of 8 µm and a transverse resolution of 20 µm, that takes 100,000 axial scans per second. A radial scan, consisting of 12 scans centered on the pupil, was conducted. Only images of sufficient quality, defined as a signal-strength index (SSI) above 3, were accepted.

**Figure 1:**
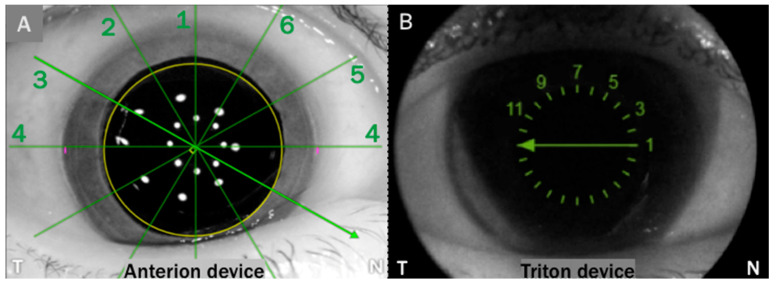
(**A**) Frontal image provided by the Anterion device, with the six axial scans numbered. (**B**) Frontal image provided by the Triton device, with the numbering of the six scans equivalent to the Anterion image. The equivalence of axial sections provided by Anterion and Triton is as follows (Anterion-Triton): 4–1 (0°), 5–3 (30°), 6–5 (60°), 1–7 (90°), 2–9 (120°), and 3–11 (150°).

Anterion provides an image with six sections, as shown in Figure 1. These scans are numbered by default, as indicated in the picture (Figure 1A). Triton offers a similar image (Figure 1B), from which we can extract the six sections equivalent to those in Anterion.

**Figure 2:**
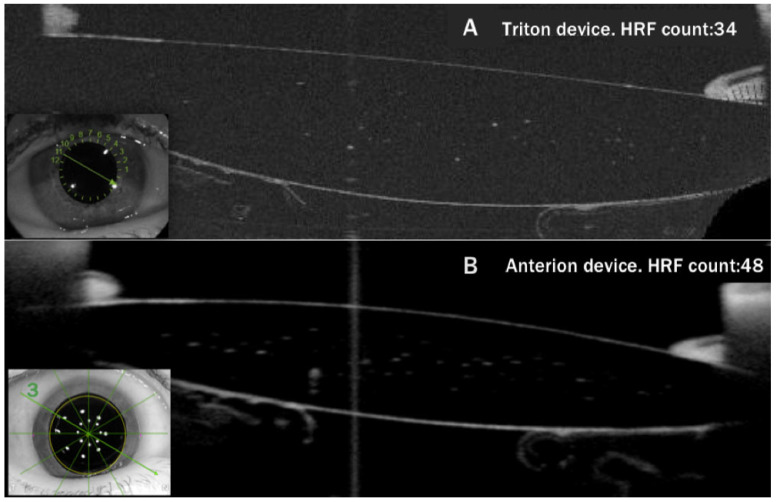
Comparison of the glistening found by Triton (**A**) and Anterion (**B**). Both images correspond to the same 26-diopter TFNT00 lens implanted 5 years ago. The manual count for the Triton image was 34 HRF, whereas for the Anterion image, B was 48 spots (both classified as glistening grade III).

Given the current lack of automated counting models that can be applied to Anterion images, glistening was evaluated by manually measuring HRF or spots by one examiner (T.S.G-P.). Initially, the six scans provided by Anterion were analyzed. Subsequently, glistening was evaluated in the six corresponding Triton scans, and this second count was performed blindly without knowledge of the results of the previous Anterion count. After counting the 3996 images (six axial scans of 333 eyes recorded using both OCT devices), the count of each Anterion image was compared with the count of the corresponding Triton image for the same eye, axis, and IOL area. In Figure 2, we can observe the manual count in the corresponding images of the same lens.

In addition to the number of HRF, the degree of glistening was classified into four categories based on the glistening severity scale recently proposed by our group [17]. Images with <5 HRF were classified as grade 0, 6 to 15 HRF as grade 1, 16 to 30 HRF as grade 2, and those with >30 HRF as grade 3. This classification was applied equally to both Anterion and Triton images.

### 2.3. Statistical Analysis

The sample size calculation was based on the primary objective of the study: to evaluate the agreement between the two SS-OCT devices in assessing glistening. An intraclass correlation coefficient (ICC) of 0.70 was anticipated, with a 95% confidence interval width of 0.20 (expected limits: 0.60–0.80). The estimation was performed assuming a 95% confidence level and a statistical power of 80%. Accordingly, the minimum required sample size was determined to be 261 eyes. To compensate for the potential exclusion of images not meeting the predefined quality criteria, a larger number of eyes was initially recruited. Ultimately, 333 eyes with images of adequate quality were included in the final analysis.

All statistical analyses were conducted using SPSS (Statistical Package for the Social Sciences, version 18.0; SPSS Inc., Chicago, IL, USA). Qualitative data are presented as frequency distributions, while quantitative data are reported as means and standard deviations or as medians and interquartile ranges (IQR), as appropriate. The normality of the variables was assessed using the Shapiro–Wilk or Kolmogorov–Smirnov tests, as applicable.

To evaluate the agreement between SS-OCT measurements, intraclass correlation coefficients (ICC) with 95% confidence intervals (95%CI) were calculated. The ICC(3,1) model was used to assess absolute agreement, and the normality of the measurements was evaluated beforehand. Bland–Altman plots were created to graphically represent the differences between measurements taken with both devices, illustrating the level of disagreement. To assess the precision and repeatability of the measurements obtained by SS-OCT, the coefficient of variation was calculated. The association between independent quantitative variables and SS-OCT measurements was evaluated using the Spearman correlation coefficient. For independent qualitative variables, the Mann–Whitney test was used to assess the association between glistening and dichotomous factors, whereas the Kruskal–Wallis test was employed for comparisons involving more than two groups, such as the analysis of glistening according to the IOL model. Simple and multiple linear regression models were used to assess whether postsurgical time acted as a confounder in the association between IOL model and glistening. A change in the regression coefficient exceeding 15% between models was considered indicative of confounding. Statistical significance was set at *p* < 0.05.

## 3. Results

This study included 285 patients with a mean age of 76.5 ± 8.0 years (range: 45–95 years). The sex distribution was 58.2% female and 41.8% male. The eyes studied were almost evenly distributed between right and left (49.4% right and 50.6% left). The median time since surgery was 1160 days (IQR 379.5–2484.5). Prior Nd:YAG laser posterior capsulotomy had been performed in 20% of the eyes analyzed, while it was absent in the remaining 80%. The implanted IOL power averaged 20.0 ± 4.3 diopters, ranging from 4 to 31 diopters.

The median number of HRF detected in a single scan was 1.1 (IQR 0.0–10.2, range 0–176) using Triton and 2.7 (IQR 0.2–20.1, range 0–250) using Anterion. The glistening grade (0–3) detected by each SS-OCT device is summarized in Table 2 (Triton) and Table 3 (Anterion). Regarding the degree of glistening, concordance was observed between the corresponding axes of the two OCT devices. The values of the Kappa index ranged from 0.4 to 0.6, with the maximum value corresponding to the 0° axis (Triton 1-Anterion 4) and the minimum value corresponding to the 60° axis (Triton 5-Anterion 6) (Table 4). This indicates moderate concordance in glistening. Of the 333 eyes across the entire study, a maximum of 3 eyes showed total disagreement in grade: either classified as grade 0 in Triton and grade 3 in Anterion or classified as grade 3 in Triton and grade 0 in Anterion.

**Figure 3:**
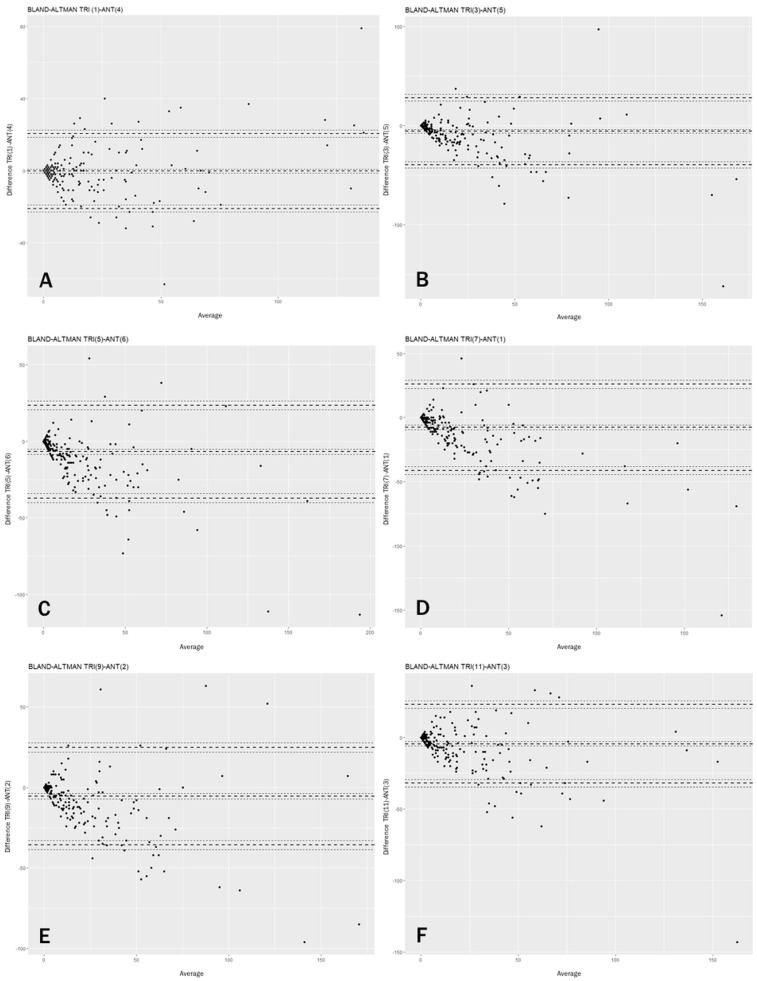
Bland–Altman plots showing the comparison between: (**A**) 0° axis (Triton 1-Anterion 4), (**B**) 30° axis (Triton 3-Anterion 5), (**C**) 60° axis (Triton 5-Anterion 6), (**D**) 90° axis (Triton 7-Anterion 1), (**E**) 120° axis (Triton 9-Anterion 2), (**F**) 150° axis (Triton 11-Anterion 3). In lenses with low glistening grades, the difference between the two devices was very low. However, at higher glistening grades, the discrepancy between both devices was greater, caused by increased variability. This interpretation applies equally to all the graphs, which reinforces the strong agreement between each pair of axes suggested by the ICC values.

**Figure 4:**
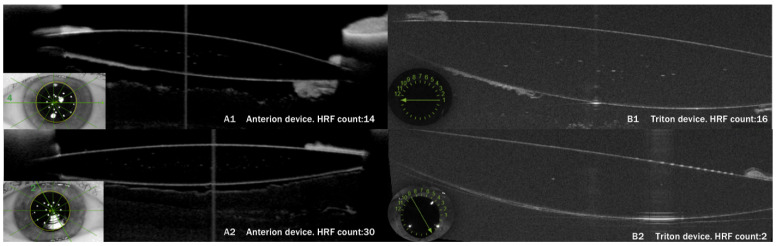
Comparison of the glistening found by Anterion (**A1**,**A2**) and Triton (**B1**,**B2**). Upper images (**A1**,**B1**) correspond to the same 25-diopter SN60AT lens and show very strong concordance between both SS-OCT devices. The lower images (**A2**,**B2**) correspond to the same 23-diopter TFNT00 lens and show very weak concordance between devices in both the HRF count and glistening grade.

The concordance of the different Anterion–Triton axes, measured using the ICC (Intraclass Correlation Coefficient) mixed models, ranged from 0.8 to 0.9, with the highest value for the 0° axis (Triton 1-Anterion 4) and the lowest for the 30° axis (Triton 3-Anterion 5 axis) (Table 5). These numbers demonstrate strong concordance between the two SS-OCT devices across all their axes. The Bland–Altman plots (Figure 3) indicate that agreement between Triton and Anterion is stronger at lower glistening levels and decreases at higher grades, due to greater dispersion of measurements. In Figure 4, an example of strong concordance in the glistening count between Triton and Anterion is shown, as well as another case of weak concordance in images from these two SS-OCT devices.

The reproducibility of the different Anterion–Triton axes, measured according to the coefficients of variation, ranged from 0.2 to 0.7, with the highest value for the 90° axis (Triton 7-Anterion 1) and the lowest for the 0° axis (Triton 1-Anterion 4) (Table 5). These results show that, although the measurements are concordant between both devices, they are not interchangeable.

Using the section with the highest concordance, in this case, the 0° axis, the relationship between glistening and various potentially related factors was analyzed. Age was found to be an independent variable from glistening in both Anterion (r = −0.1) and Triton (r = −0.009) images. This was also the case for IOL power (r = 0.2 in Anterion and r = 0.2 in Triton), sex (*p* = 0.6 in Anterion and *p* = 0.1 in Triton), and previous Yttrium-Aluminum-Garnet (YAG) laser posterior capsulotomy (*p* = 0.5 in Anterion and *p* = 0.9 in Triton). Postoperative time did show a moderate correlation with glistening (*p* < 0.001) in both the Anterion (r = 0.43) and Triton (r = 0.46) devices.

The IOL model appeared to be significantly associated with glistening in both the Anterion (*p* < 0.001) and Triton (*p* = 0.002) images. However, a notable observation emerged when the postsurgical time of each IOL group was examined (Table 6). The six IOL model groups differed significantly in postsurgical time (Kruskal–Wallis test, *p* < 0.001). This distribution closely paralleled the ranking of IOL models by glistening severity, raising the hypothesis that postsurgical time, rather than the IOL model itself, might be the true driver of HRF accumulation.

To test this hypothesis, two linear regression models were fitted for each SS-OCT device. In the simple regression model, with the IOL model as the sole predictor, the IOL model was a statistically significant predictor of HRF count for both Anterion (B = 2.263; *p* = 0.049) and Triton (B = 3.437; *p* = 0.004). However, when postsurgical time was introduced as a covariate in a multiple regression model, the effect of the IOL model lost statistical significance for both Anterion (B = 0.883; *p* = 0.42) and Triton (B = 2.024; *p* = 0.07). In contrast, postsurgical time remained highly significant in both models (B = 0.005; *p* < 0.001). Three-piece IOLs (*n* = 3) were excluded from the analysis due to small sample size.

## 4. Discussion

Several swept-source optical coherence tomography (SS-OCT) devices have been compared to analyze different ocular structures. Nevertheless, their ability to detect and quantify glistening in intraocular lenses (IOLs) has not been explored until this study. In this study, two SS-OCT devices were used to detect glistening in a large population, and our results show strong concordance between the Anterion and Triton devices, particularly for lower grades of glistening (0-II) rather than higher grades. This suggests that OCT may be more useful in ruling out glistening, as both devices consistently identify and agree on lenses that exhibit low levels of glistening.

For lenses with more than 30 spots, classified as grade III glistening, the data show weaker concordance in the precise number of hyperreflective foci (HRF) detected, with higher counts in the Anterion device. This is likely due to the high-sensitivity sensor in the Anterion SS-OCT, which, with its 14-bit capacity, can detect more than 16,000 shades of gray. Additionally, Anterion’s eye tracker enhances image precision by tracking and compensating for eye movements during the scan. This feature is crucial because even minor eye motion can compromise image quality and measurement accuracy.

It is important to note that the total number of points detected by each device has rarely been an exact match, as reflected by the reproducibility metrics. This aligns with the fact that the generated images are neither identical nor interchangeable. Nevertheless, this does not undermine the strong agreement observed, as both devices consistently detect increases or decreases in the number of HRF across different images.

Regarding the concordance between both SS-OCTs in terms of the degree of glistening, we can affirm that all corresponding axes yielded similar results, showing moderate concordance. This is related to the fact that, for instance, a count of 5 HRFs corresponds to grade 0, while a count of 6 HRFs corresponds to grade 1. Therefore, slight variations in the count could imply a change in the identified degree of glistening. Nevertheless, even taking this into account, both SS-OCTs show reasonable agreement in the detected grades, as no section revealed more than three images of complete disagreement in grading. In other words, in less than 4% of the cases, one device detected grade 0 while the other detected grade 3. In the few discrepancies observed, Triton more often detected grade 0, while Anterion detected grade 3, rather than the reverse. This pattern is consistent with the rest of our results.

Once the axis with the best concordance was identified (0° axis), it served as the basis for analyzing which patient or IOL factors might be associated with glistening. Age, sex, lens power, or the prior existence of a YAG capsulotomy have been found to be independent factors. The multivariable regression analysis showed that the IOL model was not independently associated with glistening, whereas postsurgical time emerged as the only significant predictor of HRF accumulation in both SS-OCT devices.

Regarding the IOL model, the increasingly advanced study of glistening has, in recent years, facilitated the development of so-called “glistening-free” materials. One such material is Clareon (Alcon Inc., Fort Worth, TX, USA), an acrylic copolymer composed of 2-hydroxyethyl methacrylate combined with phenylethyl acrylate as a hydrophobic component, along with an added UV filter [32,33]. Recent studies have evaluated its behavior concerning glistening formation, finding that no glistening was detected within the first year of lens implantation [17,18,19]. Our findings align with these results, as no glistening points were observed in the Clareon lenses studied, but all of them had been implanted in the year prior to the study. Therefore, our results raise an important consideration regarding the interpretation of so-called “glistening-free” materials. Since postsurgical time was identified as a confounding variable that fully accounted for the apparent differences in HRF counts across IOL models, the observed absence of glistening in newer lens designs cannot be attributed solely to their material composition. These lenses also had considerably shorter postsurgical follow-up periods compared with older models. Consequently, to establish that these materials are genuinely resistant to glistening formation, long-term follow-up studies will be essential to confirm the sustained absence of microvacuoles over time, independent of the confounding effect of postsurgical time.

To our knowledge, this study is the first to compare the Triton and Anterion SS-OCT devices. It is also the first to analyze how glistening appears on various SS-OCTs, as to date, no studies have been conducted comparing the characteristics of intraocular lenses, such as glistening. Additionally, this is the first study to analyze glistening using the Anterion device in a large population. It is the first to thoroughly examine the images provided by this device, analyzing its possible artifacts.

However, the present study has some limitations. First, in images with many points, manual assessment becomes challenging, and there is a risk of missing some of the HRFs on the lens optic. An automatic counting system would be highly advantageous for identifying glistening in these cases. In a previous study by our group [23], four lens types were evaluated using the DRI-Triton device with a deep learning algorithm, which proved highly effective for counting HRF. However, this algorithm could not be applied to the images provided by Anterion in the current study, as differences between the Anterion and Triton devices would require developing and training a new algorithm to recognize them. Therefore, within the framework of the present study, it was not feasible to compare the manual count with an automated count of glistening MVs, which underscores the need to advance in the development of specific tools for each SS-OCT device. An additional technical limitation relates to scan geometry. The detection and quantification of HRFs may be influenced by the angle of incidence of the OCT beam on the IOL surface, with optimal acquisition achieved when the beam is perpendicular to it. Although strict pupil centration and signal quality criteria were followed for both devices, perfect perpendicularity cannot be guaranteed across all radial cross-sections, and minor angular variations may affect the visibility of certain MVs. However, this effect would be expected to similarly affect both devices and should not substantially alter the concordance analysis. This factor, together with intrinsic technical differences between both SS-OCTs (including operating wavelength, axial resolution, sensitivity scale, and image processing algorithms), represents the most likely explanation for the lack of interchangeability between images obtained with both devices.

Second, this study presents a descriptive analysis of the glistening data observed in multiple IOL models. However, the small sample sizes for some groups make it difficult to perform group comparisons and draw definitive conclusions. It should also be noted that all IOL models in this study were sourced from the same manufacturer (Alcon), and the results may not be generalizable to IOLs of other materials and brands. Additionally, many patients enrolled in the study contributed with both eyes; this should be acknowledged due to the potential non-independence between fellow eyes. Likewise, this study evaluates the number of HRFs but does not consider the size or location of the points within the optic. It remains unclear whether these two factors may influence the severity or occurrence of symptoms in affected patients, which is ultimately the most critical aspect of clinical practice. Although this phenomenon has historically been considered to have little impact on visual acuity [34], it has been widely reported to be associated with visual symptoms such as glare and luminous disturbances.

Future studies are warranted to develop automated systems that can assist in counting lenses, particularly when the high density of points makes manual counting more challenging. Additionally, it would be essential to compare these deep learning algorithms to determine whether they perform equally well and yield comparable automated counts of images from both devices.

In conclusion, glistening in IOLs can be detected and quantified using two different SS-OCT devices. Both devices showed strong concordance in detecting low-grade glistening. However, for higher grades, Anterion detected significantly more HRF than Triton.

## Figures and Tables

**Table 1:** Number and type of lenses studied categorized by IOL model and manufacturer.

IOL Model	*n* (%)	Trading Company
**Non** **-toric monofocal**	**211 (63.4)**	Alcon AcrySof IQ
SN60WF	147 (44.1)	
SN60AT	54 (16.2)	
SA60AT	10 (3.0)	
**PanOptix**	**41 (12.3)**	Alcon AcrySof PanOptix
TFNT00	37 (11.1)	
TFNT40	2 (0.6)	
TFNT30	1 (0.3)	
TFNT01	1 (0.3)	
**Restor + 3**	**15 (4.5)**	Alcon AcrySof ReSTOR
SN6AD1	8 (2.4)	
SN60D3	7 (2.1)	
**Vivity**	**21 (6.3)**	Alcon AcrySof IQ Vivity
DFT015	18 (5.4)	
Vivity Toric 515	2 (0.6)	
DFT4T5	1 (0.3)	
**Toric monofocal**	**12 (3.6)**	Alcon AcrySof Toric
SN6AT6	4 (1.2)	
SN6AT5	4 (1.2)	
SN6AT4	2 (0.6)	
SN6AT3	1 (0.3)	
SN6AT2	1 (0.3)	
**Clareon**	**11 (3.3)**	Alcon Clareon
CNWTT0	10 (3.0)	
CNWTT3	1 (0.3)	
**Three-piece lenses**	**3 (0.9)**	Alcon AcrySof IQ
MN60MA with filter	2 (0.6)	
MA60MA without filter	1 (0.3)	
**Unknown model**	**19 (5.7)**	Operated in another center

IOL = intraocular lens.

**Table 2:** Glistening grade detected in axes 1, 3, 5, 7, 9, and 11 of the SS-OCT Triton.

*n* (%)	Triton 1	Triton 3	Triton 5	Triton 7	Triton 9	Triton 11
	0°	30°	60°	90°	120°	150°
Grade 0	225 (67.6)	235 (70.6)	233 (70.2)	224 (67.5)	226 (68.1)	221 (66.6)
Grade 1	40 (12.0)	38 (11.4)	42 (12.7)	53 (16.0)	44 (13.3)	46 (13.9)
Grade 2	30 (9.0)	28 (8.4)	27 (8.1)	18 (5.4)	30 (9.0)	30 (9.0)
Grade 3	38 (11.4)	32 (9.6)	30 (9.0)	37 (11.1)	32 (9.6)	35 (10.5)
Total	333 (100.0)	333 (100.0)	333 (100.0)	333 (100.0)	333 (100.0)	333 (100.0)

**Table 3:** Glistening grade detected in axes 1, 2, 3, 4, 5, and 6 of the SS-OCT Anterion.

*n* (%)	Anterion 4	Anterion 5	Anterion 6	Anterion 1	Anterion 2	Anterion 3
	0°	30°	60°	90°	120°	150°
Grade 0	225 (67.6)	199 (60.1)	194 (58.3)	197 (59.2)	201 (60.4)	198 (59.5)
Grade 1	40 (12.0)	36 (10.9)	38 (11.4)	38 (11.4)	35 (10.5)	44 (13.2)
Grade 2	30 (9.0)	44 (13.3)	40 (12.0)	36 (10.8)	40 (12.0)	39 (11.7)
Grade 3	38 (11.4)	52 (15.7)	61 (18.3)	62 (18.6)	57 (17.1)	52 (15.6)
Total	333 (100.0)	333 (100.0)	333 (100.0)	333 (100.0)	333 (100.0)	333 (100.0)

**Table 4:** The concordance between Triton and Anterion in the degree of glistening in each axis is quantified by the kappa statistic, which indicates moderate agreement across all sections. The last two columns display the total number of eyes with complete disagreement in classification, ranging from 0 to 3 eyes (0–0.9%).

Triton Axis-Anterion Axis	Kappa Index	G0 (Triton)–G3 (Anterion)	G0 (Anterion)–G3 (Triton)
Triton 1-Anterion 4 (0°)	0.6	0 (0%)	0 (0%)
Triton 3-Anterion 5 (30°)	0.5	3 (0.9%)	1 (0.3%)
Triton 5-Anterion 6 (60°)	0.4	3 (0.9%)	1 (0.3%)
Triton 7-Anterion 1 (90°)	0.5	2 (0.6%)	1 (0.3%)
Triton 9-Anterion 2 (120°)	0.5	1 (0.3%)	1 (0.3%)
Triton 11-Anterion 3 (150°)	0.5	0 (0%)	0 (0%)

G0 = grade 0; G3 = grade 3.

**Table 5:** The concordance for each corresponding pair of axes is measured by the ICC mixed models. Coefficients of variation illustrate reproducibility across the different sections of Anterion and Triton.

Triton Axis-Anterion Axis	ICC Mixed Models	Coefficient of Variation
Triton 1-Anterion 4 (0°)	0.9 (0.8–0.9)	0.2
Triton 3-Anterion 5 (30°)	0.8 (0.7–0.8)	0.7
Triton 5-Anterion 6 (60°)	0.8 (0.8–0.9)	0.7
Triton 7-Anterion 1 (90°)	0.8 (0.7–0.8)	0.7
Triton 9-Anterion 2 (120°)	0.8 (0.8–0.9)	0.6
Triton 11-Anterion 3 (150°)	0.8 (0.8–0.9)	0.6

ICC = intraclass correlation coefficient.

**Table 6:** IOL models are ranked from lowest to highest based on the presence of hyperreflective points of glistening in Anterion and Triton images (0° axis).

IOL Model	*n* IOL	Postsurgical Time (d)	Anterion HRFs	Triton HRFs
Clareon	11	47 (IQR 33–190)	0.1 ± 0.3	0.1 ± 0.3
Vivity	21	292 (IQR 144–917)	4.6 ± 11.8	2.9 ± 8.3
Toric monofocal	12	398 (IQR 343–2019)	6.3 ± 17.2	10.2 ± 20.4
Non-toric monofocal	211	1494 (IQR 543–2589)	10.3 ± 20.2	9.2 ± 20.0
PanOptix	41	1129 (IQR 801–1692)	14.3 ± 25.5	13.6 ± 28.9
Restor + 3	15	4877 (IQR 3260–6194)	30.9 ± 37.5	35.7 ± 39.5

d = days. HRFs = hyperreflective foci. IOL(s) = intraocular lens(es).

## Data Availability

The original contributions presented in the study are included in the article; further inquiries can be directed to the corresponding author.
